# Manipulating facial musculature with functional electrical stimulation as an intervention for major depressive disorder: a focused search of literature for a proposal

**DOI:** 10.1186/s12984-023-01187-8

**Published:** 2023-05-16

**Authors:** Ilya Demchenko, Naaz Desai, Stephanie N. Iwasa, Fatemeh Gholamali Nezhad, José Zariffa, Sidney H. Kennedy, Nicholas O. Rule, Jeffrey F. Cohn, Milos R. Popovic, Benoit H. Mulsant, Venkat Bhat

**Affiliations:** 1 Interventional Psychiatry Program, Mental Health and Addictions Service, St. Michael’s Hospital – Unity Health Toronto, Toronto, ON M5B 1M4 Canada; 2 Institute of Medical Science, Temerty Faculty of Medicine, University of Toronto, Toronto, ON M5S 1A8 Canada; 3 Krembil Research Institute – University Health Network, Toronto, ON M5T 0S8 Canada; 4 KITE, Toronto Rehabilitation Institute – University Health Network, Toronto, ON M5G 2A2 Canada; 5 CRANIA, University Health Network, Toronto, ON M5G 2C4 Canada; 6 Rehabilitation Sciences Institute, Temerty Faculty of Medicine, University of Toronto, Toronto, ON M5G 1V7 Canada; 7 Institute of Biomedical Engineering, Faculty of Applied Science & Engineering, University of Toronto, Toronto, ON M5S 3E2 Canada; 8 The Edward S. Rogers Sr. Department of Electrical & Computer Engineering, Faculty of Applied Science & Engineering, University of Toronto, Toronto, ON M5S 3G8 Canada; 9 Department of Psychiatry, Temerty Faculty of Medicine, University of Toronto, Toronto, ON M5T 1R8 Canada; 10 Department of Psychology, Faculty of Arts & Science , University of Toronto, Toronto, ON M5S 3G3 Canada; 11 Department of Psychology, Kenneth P. Dietrich School of Arts & Sciences, University of Pittsburgh, Pittsburgh, PA 15260 USA; 12 Campbell Family Mental Health Research Institute, Centre for Addiction and Mental Health, Toronto, ON M6J 1H4 Canada

**Keywords:** Depressive disorder, Facial muscles, Myofunctional therapy, Functional electrical stimulation, Facial expression, Neuromodulation, Interoception, Amygdala, Neurological rehabilitation, Neuroplasticity

## Abstract

**Background:**

Major Depressive Disorder (MDD) is associated with interoceptive deficits expressed throughout the body, particularly the facial musculature. According to the facial feedback hypothesis, afferent feedback from the facial muscles suffices to alter the emotional experience. Thus, manipulating the facial muscles could provide a new “mind-body” intervention for MDD. This article provides a conceptual overview of functional electrical stimulation (FES), a novel neuromodulation-based treatment modality that can be potentially used in the treatment of disorders of disrupted brain connectivity, such as MDD.

**Methods:**

A focused literature search was performed for clinical studies of FES as a modulatory treatment for mood symptoms. The literature is reviewed in a narrative format, integrating theories of emotion, facial expression, and MDD.

**Results:**

A rich body of literature on FES supports the notion that peripheral muscle manipulation in patients with stroke or spinal cord injury may enhance central neuroplasticity, restoring lost sensorimotor function. These neuroplastic effects suggest that FES may be a promising innovative intervention for psychiatric disorders of disrupted brain connectivity, such as MDD. Recent pilot data on repetitive FES applied to the facial muscles in healthy participants and patients with MDD show early promise, suggesting that FES may attenuate the negative interoceptive bias associated with MDD by enhancing positive facial feedback. Neurobiologically, the amygdala and nodes of the emotion-to-motor transformation loop may serve as potential neural targets for facial FES in MDD, as they integrate proprioceptive and interoceptive inputs from muscles of facial expression and fine-tune their motor output in line with socio-emotional context.

**Conclusions:**

Manipulating facial muscles may represent a mechanistically novel treatment strategy for MDD and other disorders of disrupted brain connectivity that is worthy of investigation in phase II/III trials.

## Introduction

Major Depressive Disorder (MDD) is a heterogeneous mental illness with complex and poorly understood pathophysiology, conceptualized as a disorder combining affective, behavioral, and cognitive symptoms [1]. In Western countries, somatic symptoms dominate the symptomatology in about two-thirds of cases [2–4]. Clinicians and researchers who have explored the association between depression and somatization have proposed that MDD is a disorder of impaired interoception and disturbed afferent bodily signals [5, 6]. In light of this hypothesis, treatments for MDD targeting disrupted interoceptive inference in emotional states, or “mind-body” interventions, have been under active investigation. Some notable examples include vagus nerve stimulation [7], botulinum toxin A (BONT-A) injections [8], and stellate ganglion block [9]. Another novel intervention based on a similar mechanistic model is functional electrical stimulation (FES), where some preliminary research has demonstrated modulatory effects on mood in healthy participants [10] and patients with MDD [11] when applied to the facial muscles involved in emotional expression.

FES is a neuromuscular stimulation technique that delivers a low-energy electrical current to skeletal muscles, causing them to contract and generate functional and purposeful movement [12]. In the literature, its function is often defined as the activation of neuromuscular units that may or may not be under voluntary control [13]. At the neural level, such activation induces changes in the afferent inputs projecting from those neuromuscular units, leading to the activation of the corresponding circuits within the central nervous system (CNS) [14–17]. The therapeutic applications of the FES have been extensively researched in stroke [18–20] and spinal cord injury (SCI) [21, 22]. FES has been used as a prosthesis to replace lost function and as a form of rehabilitation to retrain function, with the end goal of enabling a patient to execute movement without the assistance of a stimulation device. Furthermore, FES has been successfully used to restore both motor and sensory function [23], with common examples including auditory and visual neuroprostheses to reinstate hearing and vision, respectively.

Recent studies have shown that functional recovery post-FES is accompanied by plasticity in the CNS, increasing activity in regions where the activated muscles are represented topographically [14–17]. With the mechanisms of neuroplasticity being transdiagnostic [24], this narrative review introduces the basic principles of FES, provides an overview of prevailing clinical applications in psychiatry, and discusses its prospects as a tool for non-invasively altering neural circuits in MDD.

### Emotions and facial expressions in depression: four hypotheses

Facial expressions corresponding to six or more emotions (e.g., fear, anger, happiness, sadness, surprise, and disgust) are well-defined and considered universal across cultures [25–27]. These expressions can be voluntary (routed through the pyramidal motor system; i.e., motor cortex) or involuntary (routed through the extrapyramidal motor system; i.e., subcortical nuclei) [10]. The latter reflects “genuine” emotional experiences: for example, a voluntary smile without emotional input is produced for social purposes and generally involves only the zygomaticus major muscle, which raises the corners of the lips. In contrast, a spontaneous expression of positive emotion is more likely also to involve the orbicularis oculi muscles, which form “crows-feet” wrinkles at the lateral canthi of the eyes; a combination known as a “Duchenne smile” [28, 29]. Voluntarily generating and purposefully holding a facial expression has been demonstrated to be capable of eliciting a corresponding emotion [30, 31], which suggests that “mind-body” interventions modulating facial expressions may benefit individuals with disorders of disturbed affect, such as MDD.

Electromyographic studies of automatic facial expression highlight the differences between patients with MDD and healthy controls in facial expression changes characterizing particular social situations [32–34]. Research investigating facial expressions in MDD points to the attenuation of voluntary smiles produced by the zygomaticus major muscle [35, 36]. Yet, there is no consensus regarding other facial expressions: some studies report an attenuation of the facial musculature associated with negative-valence emotions [32, 36, 37], whereas others report their potentiation [38–40]. Possible explanations of these findings stem from four main hypotheses explaining facial behavior in depression: (i) the mood-facilitation hypothesis, (ii) the emotion-context insensitivity hypothesis, (iii) the social risk hypothesis, and (iv) the facial feedback hypothesis.

The **mood-facilitation hypothesis** states that affective states match the likelihood and intensity of corresponding facial expressions, with depression characterized by potentiated facial expressions in response to negative-valence states and attenuated facial expressions in response to positive-valence states [41, 42]. The **emotion-context insensitivity hypothesis**, however, postulates that depression is a defensive motivational state of disengagement from the environment; it is directed toward conserving resources by inhibiting overall emotional reactivity, which manifests in attenuated facial expressions [43]. Third, the **social risk hypothesis** views depression as a reflection on one’s engagement with the social context, particularly with threats of social exclusion [44]. Facial expressions are tailored to the social context (primarily through signaling submission and withdrawal) to protect oneself from anticipated social exclusion in the form of rejection or contempt. Thus, patients with severe depression will likely smile less and display facial expressions associated with scorn. In contrast, those with less severe depression are more likely to show signals indicating that they are willing to affiliate with the social context [34].

Another prominent theory explaining the association between facial expressions and affective states is the **facial feedback hypothesis**. Rooted in Charles Darwin’s and William James’s early views [45, 46], it posits that facial movement directly influences emotional experience [47]. Specifically, the physiological activation of facial muscles associated with expressions corresponding to specific emotions directly elicits those emotional states, whereas the lack of such activation leads to their suppression or total absence [48]. Different versions of the facial feedback hypothesis argue about the relative importance of facial feedback in the initiation of affective states [49]. The *necessity version* holds that no emotion can be experienced without facial feedback [50]. On the opposite end, the *sufficiency version* claims that facial movement alone can elicit an associated emotion [51]. Lastly, the *modulation version* views the emotional experience as elicited by some external stimulus or cue outside of one’s own facial movements, whereby a signal from facial afferents plays a modulatory role in initiating and maintaining the affective state [52].

A substantial body of work has been devoted to studying the association between facial movements and specific emotions. Human facial movements have been taxonomized by the Facial Action Coding System (FACS), with attempts to systematically categorize the physical manifestation of emotions [53]. The classical approach involves asking participants to generate specific facial expressions and record changes in self-reported emotional experiences [49]. Studies using this approach generally conclude that the induction of smile-related facial expressions leads to enhanced positive affect. In contrast, the inhibition of smile-related expressions through the activation of antagonistic muscle groups leads to its diminution [52, 54–56]. A second approach involves the presentation of emotionally charged stimuli and instructing participants to suppress induced facial movements or to constantly maintain neutral facial expressions [49]. This paradigm results in a reported decrease of both positive and negative emotions upon voluntary suppression of facial expressions and bodily movements [57–59].

### Functional electrical stimulation for major depressive disorder

The neurorehabilitative effects of FES in stroke [18–20] and SCI [21, 22], with the associated changes in the plasticity of the CNS [12, 60, 61], raise the possibility that FES could be successfully used to treat psychiatric disorders of disrupted brain connectivity, such as MDD [62]. Peripheral bottom-up activation through sensorimotor channels appears to modulate depression symptoms [63], wherein the amygdala serves as a gateway assigning emotional significance to sensory and motor events [64, 65]. Some notable examples of mood-regulatory effects of sensorimotor systems are depressive symptoms associated with inadequate vision and hearing impairment [66, 67]. Other recognized phenomena include psychomotor agitation and retardation [68, 69], stress- or emotion-induced postural adjustments and gait [70], as well as an ameliorative effect of physical exercise on mood [71].

**Fig. 1:**
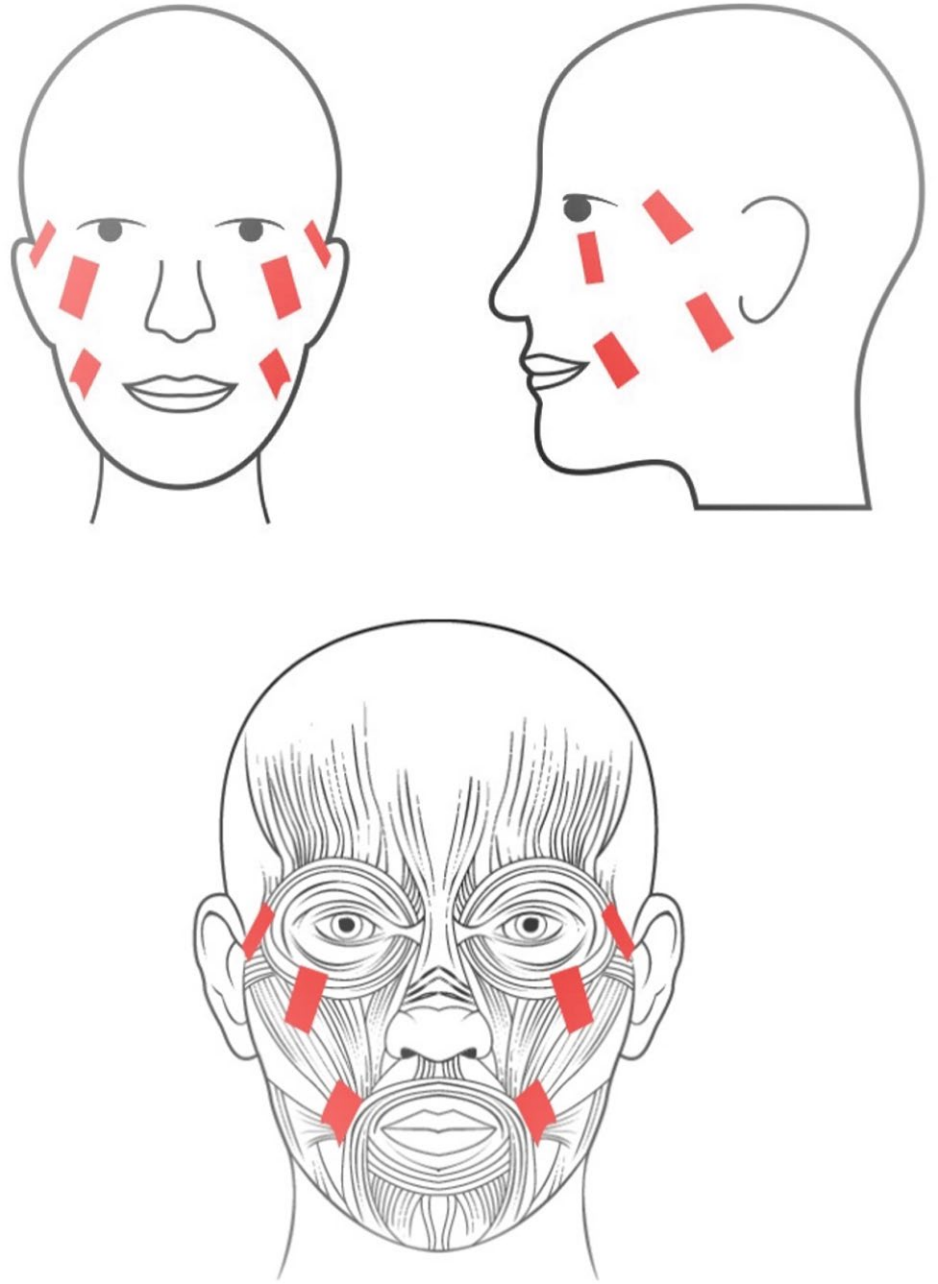
**Bilateral functional electrical stimulation of facial muscles**. Cutaneous electrode placement for the functional electrical stimulation of bilateral zygomaticus major and orbicularis oculi muscles for the treatment of major depressive disorder. Created with BioRender.com, RRID:SCR_018361.

It is hypothesized that through the activation of the zygomaticus major and orbicularis oculi muscles (Fig. 1), FES may generally upregulate the activity of sensorimotor systems due to the direct effect of electrical stimulation on muscle contraction and increase in muscle tone. In turn, this has the potential to directly facilitate neuroplastic rewiring of the primary and secondary motor cortices and modulate extrapyramidal pathways involved in the generation of involuntary motor expressions of emotions – a physiological mechanism largely informed by neurorehabilitative effects of FES in stroke and SCI [12, 60, 61]. The subcortical nuclei of the extrapyramidal system are tentatively linked to the metacognitive component of facial expressions, bridging patterns of specific motor activity with emotions [72, 73]. Given this perspective, repetitive activation of the zygomaticus major and orbicularis oculi (the “Duchenne smile” muscles) would strengthen the extrapyramidal pathway associated with positive emotions, whereas breaking the habit of activating the procerus and corrugator supercilii “frown” muscles would weaken the extrapyramidal pathway associated with negative emotions. This may lead to mood improvements and could potentially account for restoring perturbed sensorimotor balance responsible for symptoms of psychomotor retardation or agitation. In this section, we review existing evidence concerning the effect of FES on mood modulation and introduce a potential neural pathway that may be targeted by FES.

### Functional electrical stimulation modulates emotions in healthy individuals

Zariffa et al. (2014) conducted a proof-of-concept study with transcutaneous facial FES of the “Duchenne smile” muscles to examine the ability of a single FES session to modulate mood-related effects [10]. They hypothesized that FES might enhance the mood-related effects of voluntarily activating facial muscles with close neural connections to the subcortical nuclei regulating emotions, such as the amygdala. Twelve healthy participants, who received FES and were asked to voluntarily move the target muscles at the time of stimulation, were compared to 12 participants in a control group who performed the same procedure without any stimulation. Study outcomes were the scores on the Positive and Negative Affect Schedule-X (PANAS-X) [74], which asks a participant to rate 60 words or expressions describing feelings on a 1 to 5 scale according to how strongly they feel a particular emotion while completing the assessment. Those who received FES experienced changes on the “determined,” “daring,” “scared,” and “concentrating” base items of the PANAS-X, indicating that those emotions relevant to MDD could potentially be modulated by FES.

### Functional electrical stimulation improves symptoms of major depressive disorder

Kapadia et al. (2019) conducted an open-label mixed-methods study on individuals with moderate-to-severe MDD, exploring whether 10 FES sessions would lead to improvements in depressive symptoms [11]. In this study, 10 participants with MDD received FES of the “Duchenne smile” muscles three times per week. Stimulation parameters were 150 µs biphasic pulses (pulse width of the first phase), 60 Hz, with amplitude in the range of 1–15 mA, delivered for 20–25 min [10, 11]. The pulse duration and frequency were chosen during protocol development in the healthy volunteer study [10]. The pulses were asymmetric with a 150 µs leading cathodic phase. The cathode was placed over the muscles’ motor point for muscle activation. Stimulation amplitudes were adjusted at each session such that the targeted muscles achieved visible contractions with no excessive discomfort or unwanted movement (e.g., the closing of the eye). All participants received 10 sessions of FES. Because several study participants requested more FES sessions around the midpoint of the study (after 4–5 participants were already treated), the study protocol was amended to allow up to 40 FES sessions. After the amendment was introduced, the participants were invited to continue therapy for up to 40 sessions if they wished (5 of the 10 participants requested to undergo 40 FES sessions). All participants adhered to the treatment protocol.

**Fig. 2:**
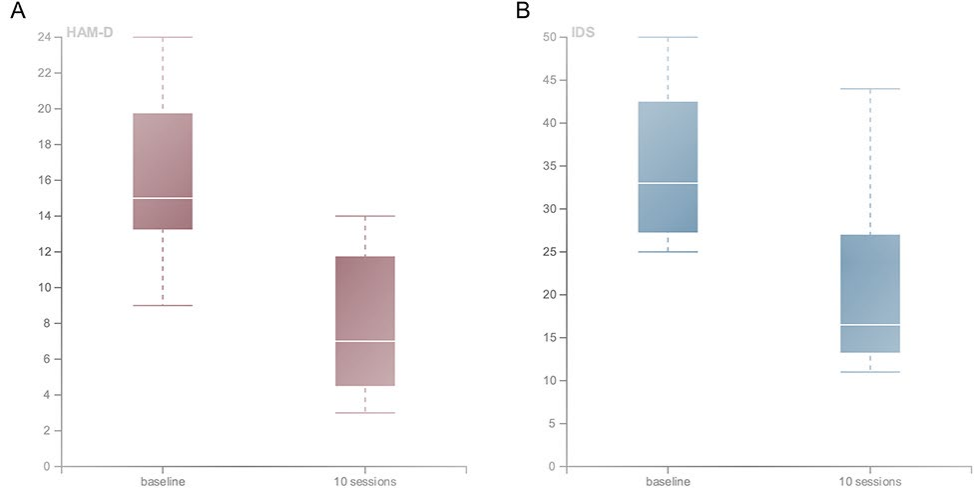
**Functional electrical stimulation improves symptoms of major depressive disorder.** Distributions of depression scores measured in participants with major depressive disorder (*n* = 10) at baseline and after 10 sessions of bilateral functional electrical stimulation (FES) of the zygomaticus major and orbicularis oculi muscles. Both the (A) Hamilton Depression Rating Scale (HAM-D, *p* = .005); and (B) Inventory of Depressive Symptomatology (IDS, *p* = .008) scores were significantly reduced post-FES. Created with RAWGraphs 2.0 beta, using the data from Kapadia et al. (2019) [11].

The results were promising: participants experienced early improvements in depressive symptoms as measured by the Hamilton Depression Rating Scale (HAM-D) [75] and Inventory of Depressive Symptomology (IDS) [76]. After completing 10 sessions, participants experienced a mean improvement on the HAM-D by 8.1 (*SD* = 5.3) points (*p* = .005) and on the IDS by 14.0 (*SD* = 11.1) points (*p* = .008) (Fig. 2**)**. Eight participants (80%) showed a reduction of at least 30%, with 5 (50%) showing clinical response (defined as a reduction in the HAM-D by at least 50%) and 6 (60%) entering remission (defined as a HAM-D total score of 7 or below). Reported adverse events were minimal and typically included redness or skin irritation underneath the stimulation site, and muscle soreness or discomfort. FES was well-tolerated, with the potential to be administered with limited clinician oversight.

### Neurobiological mechanisms and target circuitry: role of the amygdala and emotion-to-motor transformation loop

Although patterns of neural activity corresponding to facial expressions are distributed across multiple facial motor brain areas, the neurons of the amygdala appear to play a key regulatory role. The amygdala functions within the larger network organization of the human brain, sending excitatory signals to the hypothalamic-pituitary-adrenal axis, brainstem, and other limbic structures (e.g., anterior cingulate cortex, anterior insula) and receiving inhibitory projections from ventral striatum and frontal cortex [77]. The amygdala is the structure most robustly engaged in emotional processing [78–80]: functional magnetic resonance imaging (fMRI) connectivity studies have identified that it constitutes one of the key nodes within the salience and emotion network – a set of brain regions responsible for integrating sensory information to facilitate the allocation of attention toward significant stimuli, leading to behavioral decisions [64, 65]. Further, cellular studies [81, 82] have shown that an increase in the firing rate of amygdala neurons mainly occurs after the onset of the muscular activity corresponding to the movement of a facial muscle. In a common face processing network, MDD patients exhibit hyperactivation of the amygdala in response to negative stimuli and hypoactivation in response to positive stimuli, which forms mood-congruent processing bias [83].

**Fig. 3:**
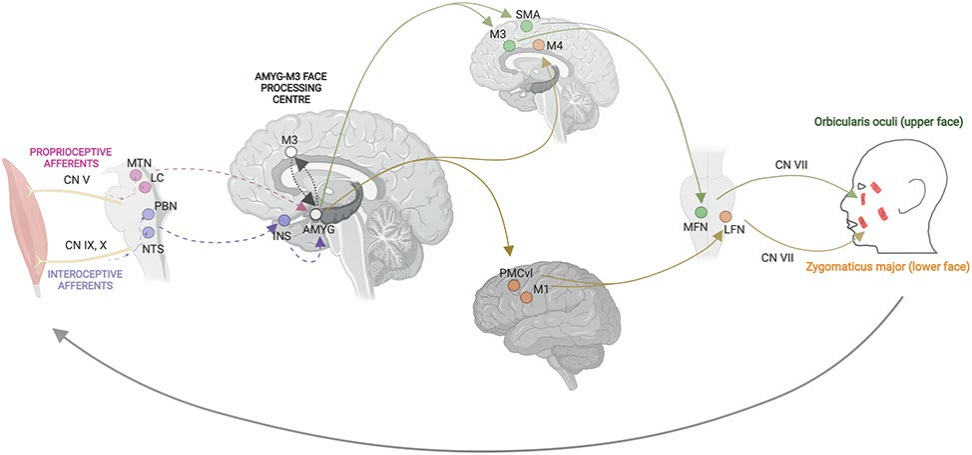
**Emotion-to-motor transformation loop**. Contraction of facial muscles relays proprioceptive (magenta) and interoceptive (purple) afferent signals to the amygdala (AMYG) via the trigeminal, glossopharyngeal, and vagus nerves and corresponding brainstem nuclei. AMYG forms a feedback loop with the anterior face area of the midcingulate cortex (M3). The AMYG-M3 connectivity establishes the processing center responsible for decision-making to select and produce a facial expression in response to a particular emotional context. These limbic inputs further calibrate the final motor output of the corticobulbar motor system, where the contraction of the upper (green) and lower (orange) face muscles is modulated via two separate anatomical pathways and the facial nerve. Dashed arrows represent afferent inputs, dotted arrows represent the processing center, and solid arrows represent efferent outputs. Abbreviations: AMYG, amygdala; CN V, cranial nerve V (trigeminal); CN VII, cranial nerve VII (facial); CN IX, cranial nerve IX (glossopharyngeal); CN X, cranial nerve X (vagus); INS, insula; LC, locus coeruleus; LFN, lateral facial nucleus; M1, primary motor cortex; M3, anterior face area of the midcingulate cortex; M4, caudal face area of the midcingulate cortex; MFN, medial facial nucleus; MTN, mesencephalic trigeminal nucleus; NTS, nucleus tractus solitarius; PBN, parabrachial nuclei; PMCvl, ventrolateral regions of the premotor cortex; SMA, supplementary motor area. Created with BioRender.com, RRID:SCR_018361.

Facial expressions are motor events generated through a sequence of reciprocal transformations between sensory and motor processes informed by interoceptive bodily afferent inputs and extracted socio-emotional significance of perceived signals [81]. Neurons of the amygdala appear to be critical in (i) the sensory monitoring of generated facial expressions, (ii) selecting an appropriate facial expression upon evaluation of the social context, and (iii) being involved in the monitoring of the facial expression of self and others through a proposed mirror neuron system [81, 84, 85]. Moreover, original research [86–89] and review articles [81, 82] point to the existence of the emotion-to-motor transformation loop (EMTL) - a limbic-motor arc that adjusts facial expressions based on the socio-emotional information coming from the environment (Fig. 3). The amygdala is the central node of EMTL, which receives input, functions as the decision-making processing center, and projects the output [24, 87, 88]. Anatomically, it forms a closed loop with the anterior face area of the midcingulate (M3) and the anterior cingulate cortex [90]. In a feedback manner, neurons of area M3 project back to the basal nuclei of the amygdala, giving rise to further feedback projections to all subdivisions of the cingulate cortex [84, 90, 91]. Other evidence points to the role of interoceptive afferents projecting to the amygdala and area M3 through the insula via the glossopharyngeal and vagus nerves [92, 93]. Therefore, the neurophysiological activity of the facial muscles induced by FES may send patterned proprioceptive and interoceptive bottom-up inputs to the amygdala through the cranial nerves and the brainstem, thus leading to neuroplastic changes in the EMTL.

With active FES applied to the zygomaticus major and orbicularis oculi “Duchenne smile” muscles, their respective contraction would potentiate proprioceptive and interoceptive afferent signals associated with a genuine smile. The proprioceptive afferents convey signals of the physical state of the agent’s face; whereas interoceptive afferents convey signals of the emotional state of the agent, signals of “social justification” to make a facial expression, and signals of self-awareness of one’s body. These afferent inputs would be relayed to the amygdala via the trigeminal, glossopharyngeal, and vagus nerves and corresponding brainstem nuclei; as well as the insula, in the case of interoceptive afferents. The feedback loop between the amygdala and area M3 establishes the processing center responsible for selecting and producing a facial expression [81, 90, 93–95]. These limbic inputs further calibrate the final motor output of the corticobulbar motor system, where the contraction of upper (e.g., orbicularis oculi) and lower face (e.g., zygomaticus major) muscles is modulated via two separate anatomical pathways, involving supplementary motor area (SMA) for the upper face and caudal face area of the midcingulate (M4) and ventrolateral premotor cortex (PMCvl) for the lower face. Motor output modified by the limbic inputs further readjusts the proprioceptive and interoceptive signals that arise from the contracted facial muscles, forming a feedback loop.

Repetitive administration of active FES may thus activate both the upper (i.e., orbicularis oculi) and lower (i.e., zygomaticus major) face muscles, which will induce a sustained “Duchenne smile” experience. Through mechanisms of Hebbian plasticity, enhancement in motor contractility of the orbicularis oculi would upregulate M3 and SMA – parts of the ascending segment of the EMTL that calibrate the motor output to upper face muscles specifically. Moreover, the prolonged experience of the “Duchenne smile” and the associated experience of positive emotions would modify afferent input to the amygdala itself with reduced neuronal activity. Together, these two processes would potentiate the EMTL pathway responsible for the conversion of associated emotional experiences to motor events, leading (through the feedback loop) to amygdala downregulation and increased functional connectivity with the nodes of EMTL and downstream networks.

The existence of EMTL is supported by studies of BONT-A paired with fMRI [48, 96]. In these studies, using BONT-A to induce paralysis of frown muscles interrupted the activity of such circuitry and dampened emotional distress signals associated with frowning in depression [48, 96]. This led to improvements in mood and decreased activity in the amygdala, with baseline activity of the amygdala restored after the paralysis had expired [96]. Evidence from these BONT-A studies supports the notion that depressive symptoms and low mood can be improved in a “bottom-up” fashion by modulating facial muscles through the amygdala circuitry, which is congruent with the neuroplastic effects of FES.

### Considerations for future studies

FES of the facial musculature is a novel experimental treatment that requires thorough investigation. To date, two preliminary studies (one in healthy individuals and one in individuals with MDD) yielded positive results regarding both the feasibility and effects of FES as a potential intervention for MDD [10, 11]. Further clinical trials are needed to demonstrate its superiority over placebo and non-inferiority over existing therapies. In regard to studying FES for MDD in future trials, one of the main goals is to optimize the number of sessions and stimulation parameters to target specific neuromuscular units with minimum discomfort and muscle fatigue. Further clinical innovations are required to make FES a more accessible therapy, such as devising a take-home FES system to make it customizable and to enable easy positioning of facial FES electrodes. Other potential avenues of research might include exploring the efficacy of regular physiotherapeutic exercises of the zygomaticus major and orbicularis oculi muscles to treat symptoms of MDD.

### Stimulation parameters

Pulse duration, current amplitude, and frequency of FES have been researched extensively and have been recently reviewed for motor retraining in individuals with neurological conditions [12, 18–20, 97–101]. For motor rehabilitation, the typically used values for muscles with intact peripheral innervation are pulse duration in the range of 150–400 µs (first phase) and the frequency of 30-50 Hz, while the exact amplitude is typically adjusted on a per-patient basis to produce a functional yet comfortable muscle contraction based on the activated target, stimulation pattern, and total duration of stimulation [10, 11, 21, 97, 101, 102].

Although the goal of motor rehabilitation using FES in individuals with stroke and SCI differs from FES for MDD (motor rehabilitation vs. mood improvements), both focus on the principles of neuroplasticity. FES, combined with a voluntary effort by the participant, results in a successful execution of the desired movement, thereby completing the afferent-efferent loop, which facilitates neuroplastic changes. Based on this hypothesis, FES may strengthen the neural connections in the primary motor cortex and amygdala, leading to improved mood-related symptoms. Preliminary studies from our laboratory have used asymmetric pulses with a 150 µs leading cathodic phase, 1–15 mA amplitude, and 60 Hz pulse frequency to stimulate facial muscles [10, 11]. Although these parameters were well-tolerated by participants, further studies are required to determine the optimal stimulation parameters for facial muscles. Importantly, factors such as the stimulator (e.g., rise time of the stimulation), electrodes, and waveforms may also affect comfort and amplitude of the delivered current required for muscle activation [102, 103].

### Dosing, adverse effects, and contraindications

Although significant work has been undertaken to understand the effects of FES in neurorehabilitation, no studies have systematically examined the optimal dosing or adverse events associated with FES. In some studies, a minimum of 20 FES sessions were needed to detect a change in function, and 40 FES sessions were required to see a difference in the quality of life [104]. No significant adverse events have been reported regarding the tolerability and safety of FES. Common mild adverse events include redness underneath the electrodes, which typically dissipates within 24 h, and occasional muscle fatigue or soreness, which also typically resolves within 24 h without intervention [101, 105]. FES has some contraindications, including metal implants at the stimulation site, pacemakers, open wounds or rash at the electrode placement site [101, 106], uncontrolled autonomic dysreflexia [107], and epilepsy [108]. Moreover, target motor neurons have to be accessible for placement of the stimulation electrodes. Finally, the patient must be cognitively able to follow the instructions and actively participate in the therapy process to obtain maximal therapeutic benefits from FES. It is thus expected that adverse events and contraindications for the FES paradigm in MDD would not differ from those characterizing FES in neurorehabilitation, despite anatomical differences in the stimulation site and motor unit size.

### Clinical innovations

Whereas pilot studies of FES therapy for MDD are encouraging, potential areas of further innovation have been identified. One of the most important concerns is the accessibility of treatment while preserving compliance. In the pilot studies, participants indicated that coming into the clinic was challenging and suggested that using a device at home would help [11]. A home-based FES device would likely facilitate access to this intervention. One challenge for a home-based FES device, particularly from a research perspective, would be the standardization of training time and electrode positioning [109]. Given the success of the in-person stimulation paradigm, one option would be to start the stimulation at the clinic for educational purposes, followed by virtually supervised home-based stimulation sessions and FES self-administration. A method to facilitate donning the electrodes in the correct positions and keeping them attached to an individual’s face would help with usability and compliance. The design of FES accessories, such as masks with customized locations for compatible novel electrodes, could eliminate the requirement for a physiotherapist to be present at every session and would also prevent the electrodes from falling off during treatment. Importantly, electrode detachment was another issue identified during FES sessions for MDD [11].

One study has demonstrated that home-based administration of another electrical stimulation modality for MDD, specifically the transcranial direct current stimulation (tDCS), was feasible when participants visited the clinic once, followed by 3 virtual sessions to get acquainted with the procedure [110]. Home-administered tDCS improved depressive symptoms; however, it was noted that computer literacy and manual dexterity requirements were limitations. In line with these observations, one recent review stated that appropriate training, the usability of the technique, appropriate ability and skills of the users, and some social interaction were all desirable for the effective use of remote electrical stimulation therapies [111]. To develop FES as a home-administered therapy, tracking the use of the device and facilitating correct electrode placements will be needed. Following the supervised use of the device, logs could be created to track its use either on paper or through mobile applications.

### Novel endophenotypes and biomarkers

The optimal frequency and duration of FES treatment required to induce acute response remain unknown, as is the course of positive therapeutic response. To better tailor FES therapy sessions to each participant, future studies shall focus on evaluating specific endophenotypes of MDD that characterize and predict treatment response to FES. One of the approaches could be using the Research Domain Criteria (RDoC) framework [112], with the effects of FES assessed in a pre- and post-treatment fashion on selected metrics corresponding to the 6 functional domains of negative valence, positive valence, cognition, social processes, arousal and regulatory processes, and sensorimotor processes. For instance, RDoC studies could include psychometric, behavioral, neuroimaging, physiological, and molecular metrics to provide mechanistic insight into how FES leads to changes in mood, how the EMTL and its nodes are modulated, and which patients may benefit from FES. In the long run, predictive biomarkers would facilitate the development of preventive FES therapy with optimal duration and frequency that patients could self-deliver at home. It would also prompt a better understanding of the progression of MDD post-FES, leading to personalized FES therapies.

## Conclusions

Rooted in facial feedback and neuroplasticity theories, FES is a promising novel intervention for MDD with established safety, feasibility, and practicality. This narrative review summarizes a theoretical foundation behind the link between facial expressions and depression, reviewing evidence supporting the use of repetitive FES for MDD and its putative mechanisms of action. The use of FES for MDD is supported by feasibility and preliminary positive results. However, this area of research now requires the design and development of further phase II/III clinical trials that would focus on comparing its superiority over placebo (e.g., sham FES) and non-inferiority over other interventions. If proven efficacious, transcutaneous facial FES therapy could offer an alternative neuromodulation-based treatment modality for MDD or other psychiatric disorders of disrupted brain connectivity, which would pose a minimal-to-no risk of adverse effects and would be easy to administer.

## Data Availability

Not applicable to this review article as no datasets were generated or analyzed.

## Abbreviations

AMYG: Amygdala
CN IX: Cranial nerve IX (glossopharyngeal)
CN V: Cranial nerve V (trigeminal)
CN VII: Cranial nerve VII (facial)
CN X: Cranial nerve X (vagus)
CNS: Central nervous system
FES: Functional electrical stimulation
fMRI: Functional magnetic resonance imaging
HAM-D: Hamilton Depression Rating Scale
IDS: Inventory of Depressive Symptomatology
INS: Insula
LC: Locus coeruleus
LFC: Lateral facial nucleus
M1: Primary motor cortex
M3: Anterior face area of the midcingulate cortex
M4: Caudal face area of the midcingulate cortex
MDD: Major depressive disorder
MFN: Medial facial nucleus
MTN: Mesencephalic trigeminal nucleus
NTS: Nucleus tractus solitarius
PANAS-X: Positive and Negative Affect Schedule-X
PBN: Parabrachial nuclei
PMCvl: Ventrolateral regions of the premotor cortex
RDoC: Research Domain Criteria
SCI: Spinal cord injury
SMA: Supplementary motor area
tDCS: Transcranial direct current stimulation
